# A retrospective claims analysis: Compliance and discontinuation rates among Canadian patients with multiple sclerosis treated with disease-modifying therapies

**DOI:** 10.1371/journal.pone.0210417

**Published:** 2019-01-14

**Authors:** Pierre Duquette, Michael Yeung, Soukaïna Mouallif, Hamid Reza Nakhaipour, Paola Haddad, Robyn Schecter

**Affiliations:** 1 Notre-Dame Hospital, Université de Montréal, Montreal, Quebec, Canada; 2 University of Calgary Multiple Sclerosis Clinic, Calgary, Alberta, Canada; 3 Novartis Pharmaceuticals Canada Inc., Dorval, Quebec, Canada; University of Oxford, UNITED KINGDOM

## Abstract

**Background:**

Compliance to disease modifying therapy (DMT) is associated with a reduced risk of relapse, lower healthcare resource utilization, and improved health-related quality of life in patients with multiple sclerosis (MS). Our objective was to assess the compliance and discontinuation rates of fingolimod relative to other oral, injectable, and infusible DMTs available on the market at the time of the study in Canada in patients with relapsing—remitting MS (RRMS).

**Methods and findings:**

We conducted a retrospective claims analysis. Patients with RRMS with ≥ 1 prescription for each DMT were included. Compliance (medication possession ratio of ≥ 80%) and discontinuation (gap > 30 days from the end of the index prescription) were calculated at the 6-, 12- and 24-month time points. Compliance with fingolimod at the 6-, 12- and 24-month time points was 75%, 75% and 70%, respectively; compared with DMF [70% (P < 0.001), 68% (P < 0.001), and 56% (P < 0.001), respectively], and BRACE [53% (P < 0.001), 47% (P < 0.001), and 35% (P < 0.001), respectively]. Compliance with fingolimod was comparable to teriflunomide at each time point, but was higher compared to natalizumab [70% versus 57% (P < 0.001)] at the 24-month time point. At the 6-, 12- and 24-month time points, patients on fingolimod had the lowest discontinuation rate (26%, 24%, and 29%, respectively) compared to BRACE [49% (P < 0.001), 44% (P < 0.001), and 57% (P < 0.001)], respectively], and natalizumab [33% (P < 0.001), 29% (P < 0.001), and 45% (P < 0.001), respectively], and was similar to teriflunomide (26%, 25%, and 31%, respectively).

**Conclusions:**

The compliance rate in fingolimod treated patients at the 24 month time point was higher than that observed in natalizumab treated patients. The discontinuation rate was lower with fingolimod compared to other DMTs at all time points but was similar to teriflunomide.

## Introduction

Multiple sclerosis (MS) is the most common disabling chronic neurodegenerative disorder. As of 2013, the estimated global prevalence of MS was 2.3 million, and approximately 85% of MS patients had relapsing—remitting MS (RRMS) at diagnosis [1]. Canada is a country with a high prevalence of MS, and an estimated 100,000 Canadians are affected by this disease [2]. Currently, there is no cure for MS, and treatment mainly focuses on recovering rapidly from MS attacks, slowing disease progression, and managing MS symptoms. Disease modifying therapies (DMTs) are efficacious in reducing the frequency and severity of relapses and/or delaying disability progression in MS patients [3]. A total of 14 DMTs have been approved for use in Canada for the management of RRMS, namely, teriflunomide (Aubagio), interferon beta-1a (Avonex, Rebif), interferon beta-1b (Extavia, Betaseron), glatiramer acetate (GA) (Copaxone, Glatect), fingolimod (Gilenya), alemtuzumab (Lemtrada), peginterferon beta-1a (Plegridy), dimethyl fumarate (DMF;Tecfidera), natalizumab (Tysabri), cladribine (Mavenclad), and ocrelizumab (Ocrevus). Of these, interferons, glatiramer acetate, teriflunomide and dimethyl fumarate are generally used as first line therapies, while, fingolimod, cladribine, ocrelizumab, alemtuzumab and natalizumab as second line therapies [4]. The efficacy and effectiveness of these DMTs vary, and are directly dependent on patient adherence [5].

The International Society for Pharmacoeconomics and Outcomes Research (ISPOR) medication compliance and persistence work group developed definitions for compliance and persistence. According to ISPOR, compliance is synonymous to adherence and is defined as “the extent to which a patient acts in accordance with the prescribed interval and dose of a dosing regimen”. Persistence is defined as “the duration of time from initiation to discontinuation of therapy” [6]. Compliance to DMTs leads to a reduced risk of relapse [7, 8], lower healthcare resource utilization [8, 9], reduced incidence of MS-related hospitalization [10], improved health-related quality of life [11, 12], and significant savings in direct and indirect costs among MS patients [9]. In contrast, suboptimal adherence to DMTs among patients with MS has been associated with poor patient outcomes and higher cost of care [10]. This further leads to either switching or discontinuation of therapy and has been associated with a higher risk of relapse and disability progression [13].

Self-injectable DMTs have a high discontinuation rate and poor compliance; oral DMTs such as fingolimod have shown higher compliance and lower discontinuation rates compared to self-injectable DMTs [5, 13–15] and other oral DMTs [16]. The common reasons for discontinuation of injectable DMTs include flu-like symptoms, injection site reactions, dosing regimen, needle phobia, duration of disease and treatment, patient perception of medication risk and benefit, and burden associated with the treatment [8, 17]. In order to assess real-world compliance and discontinuation of fingolimod relative to other oral, injectable, and infusible DMTs that were available on the market at the time of the study in Canada in patients with RRMS, we conducted a retrospective claims analysis based on the IQVIA Rx Dynamics database.

## Materials and methods

### Study design and data source

This was a retrospective analysis of claims data from patients who were covered by private insurers and had at least one prescription filled for a DMT for RRMS. Claims data were collected for each DMT (oral: Gilenya (fingolimod), Tecfidera (DMF), and Aubagio (teriflunomide); infusible: Tysabri (natalizumab); and injectables: **BRACE** therapies include **B**etaseron (interferon beta-1b), **R**ebif (interferon beta-1a), **A**vonex (interferon beta-1a), **C**opaxone (Glatiramer acetate, GA), and **E**xtavia (interferon beta-1b). Each claim represents an individual patient and patients were categorized into a cohort based on a prescription that was filled.

Patients who were new to the DMT were selected in the first month of the study and tracked at 6-, 12- or 24-months to determine their compliance and discontinuation rates. New to DMT patients were defined as: i) Treatment naive/first-line; ii) Switched from another DMT; iii) Lapsed users—when patients make a claim for a new DMT X after a lapse of 365 days or more of taking DMT Y; and iv) Restart—when the patient stops a DMT for at least 365 days and returns on the same DMT.

For compliance and discontinuation at 12- and 24-months, only those patients who made a claim for a DMT during the final study month or in the following three months were included. Patients who did not make a claim for any DMT during the final study month or in the following three months were counted as a loss to follow-up.

For these analyses, claims data from patients who were covered by private insurers were accessed through Rx Dynamics from IQVIA Solutions Canada Inc., which provides an accurate measure of longitudinal drug utilization within Canada in selected therapeutic markets. On a monthly basis, it tracks and reports the number of new or repeat or total patients, type of prescription (switch, first-line, or add-on), demographics, compliance, and discontinuation for all DMTs [18].

### Outcomes measured

The compliance for each cohort of claims for a specific DMT was measured using medication possession ratio (MPR), which is defined as the number of doses dispensed in relation to the dispensing period [6, 19].

$$\text{MPR}=\frac{\text{Total}\;\text{number}\;\text{of}\;\text{days}\;\text{supplied}\;\left(\text{actual}\;\text{usage}\;\text{days}\right)}{\text{Total}\;\text{number}\;\text{of}\;\text{patients}\;\text{in}\;\text{the}\;\text{cohort}\times \text{number}\;\text{of}\;\text{days}\;\text{in}\;\text{the}\;\text{study}\;\left(\text{ideal}\;\text{usage}\;\text{days}\right)}$$

The cohort of claims for each DMT was considered compliant if the MPR was ≥ 80%. This calculation does not eliminate non-retained days, wherein a patient has switched to a different product or stopped the therapy. Discontinuation was defined as a gap in therapy when a subsequent prescription for the index treatment occurred > 30 days from the end of the previous prescription. The discontinuation rate was calculated based on patients who stopped therapy within a 60-day period or who were switched to another DMT within the same period.

The 6-, 12- and 24-month cohorts of patient claims for each DMT that were available on the market at the time of the study in Canada from January 2013 to January 2017, were used for this analysis. As specified above, we used three closed time cohorts of 6-, 12- and 24-months. For these cohorts, the concept of “follow-up” does not apply because patient claims were only included in the analyses if the data was available for the entire time period for each cohort. Compliance and discontinuation rates were measured at 6-, 12- and 24-months from the start of the DMT.

### Statistical analysis

A Chi-square test was used to compare fingolimod relative to other DMTs in terms of the percentage of patients deemed compliant, and the percentage of patients who discontinued. Since the data for the analysis was obtained from claims datasets, the baseline patient characteristics are not available. All analyses were conducted in SAS version 9.1.

This study complied with all applicable laws, regulations, and guidance regarding patient protection, including patient privacy. All data were compliant to the Health Insurance Portability and Accountability Act (HIPAA). This was a retrospective study analyzing an unidentified administrative claims database; therefore, it was exempt from review by a Research Ethics Board (REB).

## Results

**Fig 1:**
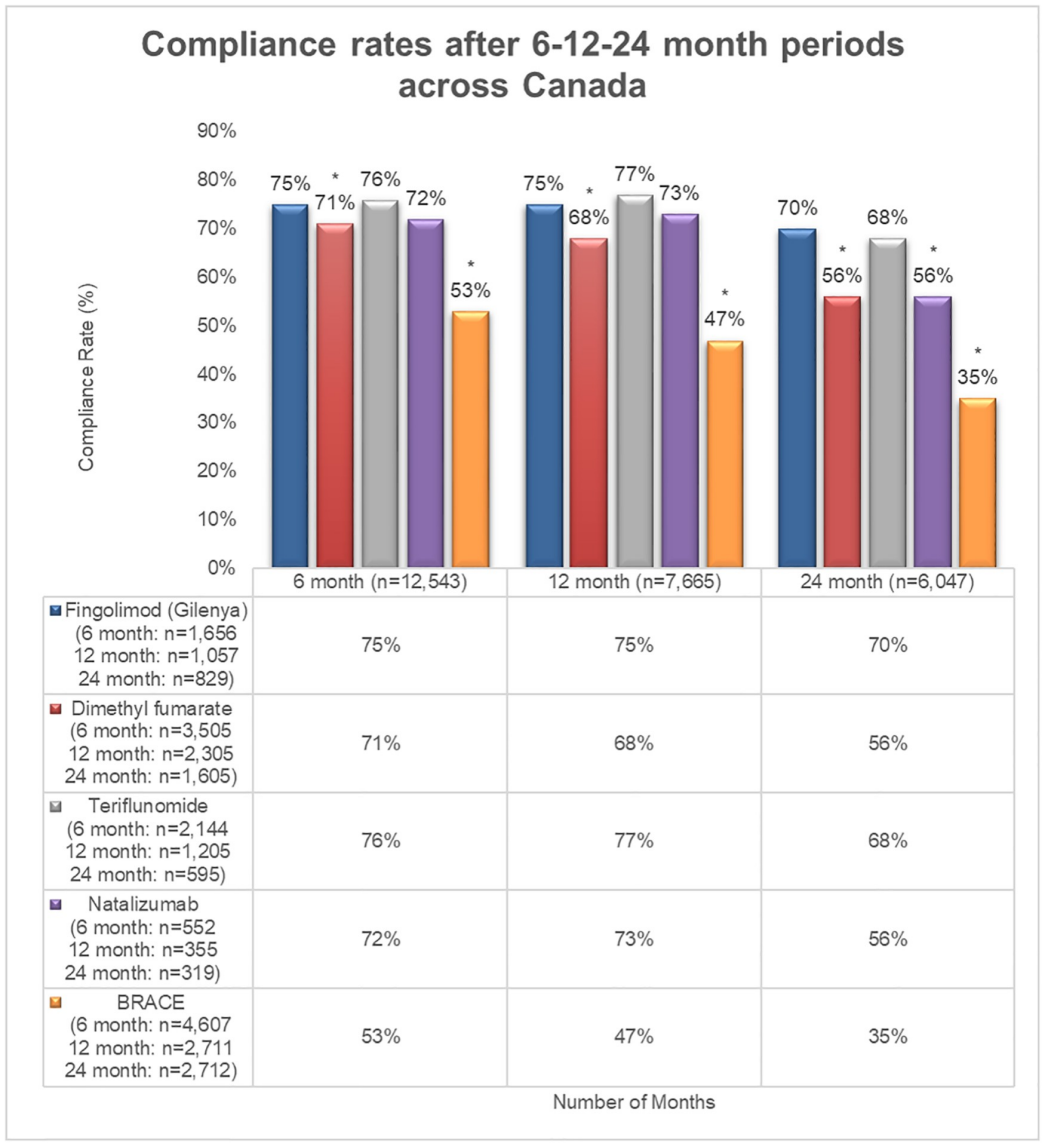
Rate of compliance at the 6-, 12- and 24-month time points. *indicates significant difference vs. fingolimod at P < 0.001; BRACE: Betaseron, Rebif, Avonex, Copaxone, and Extavia.

The total number of patients included in the compliance and discontinuation cohorts were 26,157 and 32,795, respectively. Compliance and discontinuation data were collected at the 6-month (n = 12,464, n = 13,640, respectively), 12-month (n = 7,633, n = 10,754, respectively), and 24-month (n = 6,060, n = 8,401, respectively) time points. A higher proportion of patients treated with fingolimod were deemed compliant at the 6-, 12- and 24-month time points compared to DMF, and BRACE. Compliance at the 6-, 12- and 24-month time points with fingolimod was 75%, 75%, and 70%, respectively, compared to DMF (70%, 68%, and 56% [all P < 0.001]) and BRACE (53%, 47%, and 35% [all P < 0.001]). Compliance with fingolimod was comparable to teriflunomide at each time point (76%, 76%, and 68%, respectively) and natalizumab at the 6- and 12-month time points (72%, 73%, respectively) (Fig 1). However, a higher proportion of patients treated with fingolimod were compliant at the 24 month time point compared to natalizumab (70% versus 57% [P < 0.001]).

At the 6-, 12- and 24-month time points, patients on fingolimod had a lower discontinuation rate (26%, 24%, and 29%, respectively) compared to BRACE (49%, 44%, and 57% [P < 0.001], respectively), and natalizumab (33%, 29%, and 45% [all P < 0.001], respectively). However, the discontinuation rate for fingolimod was similar to teriflunomide (26%, 25%, and 31%, respectively) (Fig 1).

**Fig 2:**
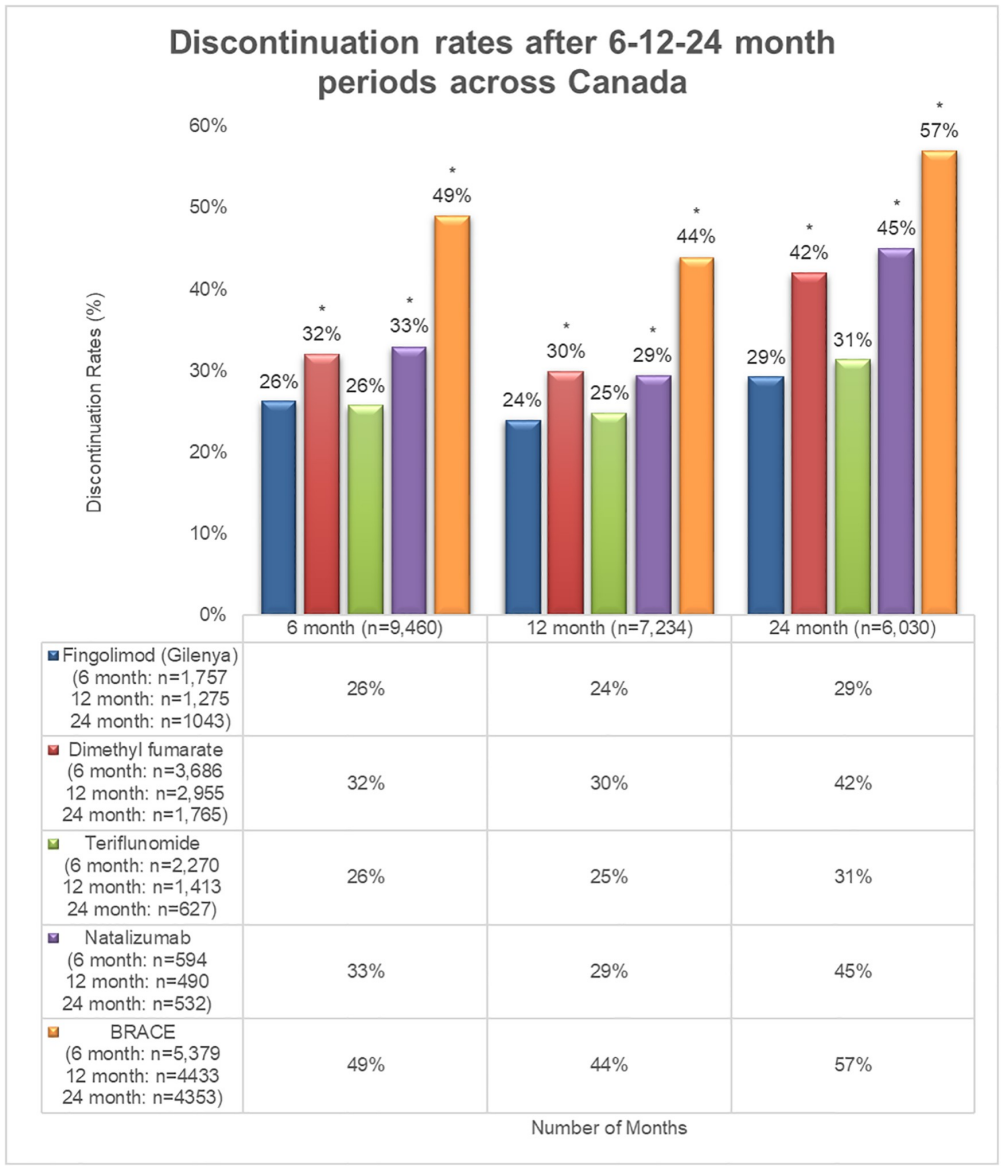
Rate of discontinuation at the 6-, 12- and 24-month time points. *indicates significant difference vs. fingolimod at P < 0.001; BRACE: Betaseron, Rebif, Avonex, Copaxone, and Extavia.

The fingolimod and teriflunomide cohorts had the lowest rates of discontinuation at the 6 and 12 month time points, while the fingolimod cohort had the lowest rate of discontinuation at the 24 month time point. Patients on BRACE therapies had the highest discontinuation rate at all time points (Fig 2).

## Discussion

Over the past decade, several studies have assessed the comparative compliance or adherence and discontinuation rates among injectable DMTs. Owing to injection-site reactions and limited effectiveness, patients were dissatisfied and showed limited adherence. Infusible DMTs are more efficacious than injectable DMTs but are associated with substantial safety concerns. These limitations led to the discovery and development of oral DMTs. Currently, there are four oral DMTs (fingolimod, teriflunomide, DMF and cladribine) approved for treating RRMS as an alternative to injectable DMTs. The efficacy of fingolimod is considered to be superior to that of injectable DMTs [20, 21], and it is associated with a higher rate of compliance and lower discontinuation rate compared to other oral, injectable and infusible DMTs [14–16]. This analysis provides the comparative insight into short- and medium-term compliance and discontinuation of fingolimod relative to other oral, injectable and infusible DMTs that were available on the market in Canada at the time of the study.

In this analysis, the most commonly used measure of compliance, MPR, was determined using a variable-interval approach [22]. This approach primarily focuses on a patient’s commitment to the prescribed dosing intervals. Patients treated with the oral DMTs like fingolimod and teriflunomide showed better compliance and lower discontinuation rates compared to those treated with other DMTs. This analysis confirms previous findings from US studies that fingolimod has better compliance and discontinuation rates compared to injectable DMTs and natalizumab [14, 15]. A retrospective claims study in the US showed that the 12-month compliance and discontinuation rate favored fingolimod relative to BRACE. A MPR of ≥ 80% was observed in 90.5% of the fingolimod cohort previously treated with other DMTs (experienced cohort) and in 87.4% of the DMT-naïve cohort. Overall, 31.3% of patients from the DMT-naïve cohort and 25.7% from the experienced cohort discontinued fingolimod [14]. Another retrospective study from the US reported a lower rate of non-adherence and discontinuation in patients on fingolimod compared to patients on injectable (interferon, GA) and infusible DMTs (natalizumab) [15]. The proportion of non-adherent patients (MPR < 80%) over 360 days of follow-up was the lowest in patients on fingolimod (6.2%), followed by patients on natalizumab (11.3%), interferon (11.9%), and GA (11.9%). In comparison to fingolimod, both injectable DMTs and natalizumab showed a 2-fold increase in the risk of non-adherence. The rate and risk of discontinuation were significantly higher for patients on interferon, GA, and natalizumab [15]. A recent real-world study showed better adherence and persistence with fingolimod compared with other oral DMTs over a period of 12 months. Compared to fingolimod, patients on DMF and teriflunomide were significantly less likely to have an MPR ≥ 80% and PDC (percentage of days covered) ≥ 80%, and the odds of discontinuation were twice as high [16]. In most Canadian provinces, records of prescription data are collected only for special population groups similar to those covered by publicly funded drug plans, and the extent of the coverage differs across provinces. In this study, private claims data of patients with RRMS were accessed through Rx Dynamics that provides an accurate measure of longitudinal drug utilization within Canada in selected therapeutic markets. On a monthly basis, it tracks and reports the number of new/repeat/total patients, source of business (switch, first-line, or add-on), demographics, retention, and daily dose for each DMT [18].

One of the limitations of these analyses is that not all eligible patients were captured as the IQVIA RX Dynamics database includes only private insurer claims. Without the actual knowledge of medication consumption by the patient, our compliance assumptions were based on renewal prescriptions. It would be informative to collect compliance information directly from the patient which is an area for future research. Understanding the impact of the mode of administration or injection frequency on adherence, and evaluating the direct impact of the patient out-of-pocket costs are required to confirm the findings of this analysis. Since this is a claims database analyses and data are not collected for the purpose of research, information was unavailable for potential confounding variables e.g. age, clinical outcomes including disease severity and duration. Another limitation of this study was that the cohorts were not differentiated based on their previous treatments as treatment-naïve and experienced. This may affect the compliance and discontinuation rates in the different cohorts. Finally, the limitation due to inaccurate coding in the claims database cannot be ruled out.

In conclusion, this retrospective analysis showed that a higher percentage of patients with RRMS treated with fingolimod or teriflunomide were deemed compliant compared to those treated with other DMTs after 24 months. At the 6-, 12- and 24-month time points, patients treated with fingolimod or teriflunomide had the lowest discontinuation rate compared to either BRACE, natalizumab, and DMF, respectively. When interpreting the findings from this research, an important consideration is the line of therapy which was investigated. Fingolimod and natalizumab are generally used as 2^nd^ line DMTs, whereas the other treatments are generally used as 1^st^ line DMTs. Compliance and discontinuation parameters of various DMTs should factor into treatment decision making when a sequencing plan is being developed for a given patient.

Improved compliance may help achieve therapeutic goals and may be associated with improved clinical benefits. These findings may help MS management strategies, which may lead to improved clinical and economic outcomes.
